# Society Meetings

**Published:** 1905-08

**Authors:** 


### SOCIETY MEETINGS.

The American Association of Obstetricians and Gynecologists will hold its eighteenth annual meeting at the Hotel Astor, Longacre Square, New York, Tuesday, Wednesday, and Thursday, September 19, 20, and 21, 1905.

Dr. Robert T. Morris, 616 Madison avenue, chairman, Dr. Samuel W. Bandler, 229 West 97th street, and Dr. James N. West, 71 West 49th street, constitute the local committee of arrangements, one or all of whom will gladly furnish information to members and guests upon application.

The following is a list of papers offered up to the present date:

The president's address, Howard Williams Longyear, Detroit.

1. Title to be announced, J. H. Carstens, Detroit.
2. Title to be announced, Magnus A. Tate, Cincinnati.
3. Personal experience in hysterectomy for myofibroma of the uterus, Miles F. Porter, Fort Wayne.
4. Title to be announced, J. W. Hyde, Brooklyn.
5. Diagnosis, John B. Deaver, Philadelphia.
6. Treatment of procidentia uteri, H. E. Hayd, Buffalo.
7. Perineal injuries and methods of repair, Joseph Price, Philadelphia.
8. Title to be announced, H. C. Pantzer, Indianapolis.
9. Appendicitis as a factor in the diagnosis of abdominal and pelvic diseases, Rufus B. Hall, Cincinnati.
10. Title to be announced, W. A. B. Sellman, Baltimore.
11. Indications for hysterectomy in puerperal eclampsia, Charles G. Cumston, Boston.
12. Title to be announced, Edwin Walker, Evansville.
13. Title to be announced, John Young Brown, Saint Louis.
14. Papillary cystadenoma of the breast, Edward J. Ill, Newark.
15. Normal saline solution and its application to conditions, Walter B. Dorsett, Saint Louis.
16. Colon bacillus leucorrhea, Robert T. Morris, New York.
17. Gallstones in the cystic duct, L. H. Dunning, Indianapolis.
18. Title to be announced, O. H. Elbrecht, Saint Louis.
19. Pelvic infection—etiology; routes of invasion; pathologic changes and clinical courses, John B. Murphy, Chicago.
20. Surgery of the liver, William J. Gillette, Toledo.
21. The treatment of puerperal eclampsia, E. Gustav Zinke, Cincinnati.
22. The evolution of the anterior transplantation of the round ligaments for uterine displacements, A. H. Ferguson, Chicago.

23. Title to be announced, H. Howitt, Guelph.
24. Myomectomy, W. P. Manton, Detroit.
25. Title to be announced, Thomas B. Eastman, Indianapolis.
26. Extraperitoneal pregnancy; with report of a case, Charles A. L. Reed, Cincinnati.
27. Title to be announced, F. F. Simpson, Pittsburg.
28. Title to be announced, L. S. McMurtry, Louisville.
29. Some general principles in conservative pelvic surgery, J. F. W. Whitbeck, Rochester.
30. Title to be announced, Charles L. Bonifield, Cincinnati.
31. Title to be announced, John D. S. Davis, Birmingham.
32. Title to be announced, X. O. Werder, Pittsburg.
33. Title to be announced, B. Sherwood Dunn, Easton.
34. Observations respecting treatment of face presentations, A. P. Clarke, Cambridge.
35. Title to be announced, C. C. Frederick, Buffalo.

All members of the medical profession are cordially invited to attend the scientific sessions.

Howard Williams Longyear, president; William Warren Potter, secretary.

---

The Buffalo Academy of Medicine, through its president and secretary, has issued an invitation to the members to contribute papers for the sessions of 1905-6. Titles of papers should be sent to the secretary, Dr. Edwin A. Bowerman, 237 East Utica street.

---

The National Association of United States Pension Examining Surgeons held its fourth annual meeting at Chicago, July 7 and 8, 1905. Dr. Nelson W. Wilson, of Buffalo, read a paper on Diseases of the genitourinary tract, which will be published in a later issue of the Journal.

---

The Lake Keuka Medical Association held its annual meeting at Grove Springs, July 13 and 14, 1905, under the presidency of Dr. C. W. M. Brown, of Elmira. Drs. Mann, Frederick, and Hopkins, of Buffalo, were announced to participate in the proceedings.

---

The Lake Erie Medical Society held its regular quarterly meeting at North Collins, July 21, 1905. Dr. Grover Wende, of Buffalo, read a paper on Röntgen therapy—its sphere of application in the treatment of skin diseases. Dr. Irving M. Snow, of Buffalo, read a paper on Infections of new born infants.

